# A Proteomics Sample Preparation Method for Mature, Recalcitrant Leaves of Perennial Plants

**DOI:** 10.1371/journal.pone.0102175

**Published:** 2014-07-16

**Authors:** Deng Gang, Zhong Xinyue, Zhang Na, Lao Chengying, Wang Bo, Peng Dingxiang, Liu Lijun

**Affiliations:** 1 MOA Key Laboratory of Crop Ecophysiology and Farming System in the Middle Reaches of the Yangtze River, College of Plant Science and Technology, Huazhong Agricultural University, Wuhan, Hubei, China; 2 School of Agricultural Science, Yunnan University, Kunming, Yunnan, China; 3 Wuhan Agriculture and Science Institute, Huangpi District, Wuhan, Hubei, China; Lawrence Berkeley National Laboratory, United States of America

## Abstract

Sample preparation is key to the success of proteomics studies. In the present study, two sample preparation methods were tested for their suitability on the mature, recalcitrant leaves of six representative perennial plants (grape, plum, pear, peach, orange, and ramie). An improved sample preparation method was obtained: Tris and Triton X-100 were added together instead of CHAPS to the lysis buffer, and a 20% TCA-water solution and 100% precooled acetone were added after the protein extraction for the further purification of protein. This method effectively eliminates nonprotein impurities and obtains a clear two-dimensional gel electrophoresis array. The method facilitates the separation of high-molecular-weight proteins and increases the resolution of low-abundance proteins. This method provides a widely applicable and economically feasible technology for the proteomic study of the mature, recalcitrant leaves of perennial plants.

## Introduction

The study of proteomes is based on the qualitative and quantitative identification of proteins, their intracellular localizations and their interactions through separation and identification. The objects of study are usually total protein lysates or a sub-fraction thereof from cells, tissues or organs [1]. Cells maintain homeostasis through different protein functions. Alterations in environmental conditions (pathology, drought stress, salt stress, etc) result in differential accumulation of proteins. Therefore, the identification of these alterations in protein accumulation or expression can provide important information for the study of related physiological processes [2].

Two-dimensional gel electrophoresis (2-DE) is commonly used for the separation of thousands of proteins from plant tissues [3]. The success of proteomics studies on different organs and plants depends on the protein sample preparation of the materials [4]. This is especially important for differential proteomics, which focuses on the slight differences in protein abundance between treatment and control groups, the selection of an appropriate method is key for obtaining reliable experimental results [5]. The wide range of biochemical properties of proteins (such as isoelectric point, expression abundance, solubility etc) can compromise the extraction of the full proteome depending on the specific extraction method. Thus, there are few sample preparation methods that can be used simultaneously in different species and organs [2]. Plant cells contain large quantities of nonprotein substances such as polysaccharides, lipids, and organic acids [6]. While the plant cell wall is comprised of large amounts of cellulose and pectin and can have a rigid secondary cell wall due to lignification of mature cells. These substances have a significant influence on the quality of protein extracts and consequently on the results of two-dimensional gel electrophoresis [7], [8], [9].

Optimal protein sample preparation is required to efficiently remove nonprotein substances from the sample tissues, and methods must be adapted to different plant organs and species [4]. However, the sample preparation methods currently in common use are often not applicable to a range of plants and tissue. Sample preparation for proteomics is often applied to young and tender plant tissues [10]; the preparation of mature organs is relatively rare. Reports on the application of two-dimensional gel electrophoresis to mature tissues do exist, but the research is mainly focused on annual plants, including the mature seeds of *Lupinus albus* [11], *A. thaliana* [12], *Arachis hypogaea* [13], and *Triticum aestivum* [14], [15]; the mature leaves of *Lathyrus sativus* [16] and *Oryza sativa* [17]; and the mature pollen of *A. thaliana* [18], *Oryza sativa* [10], and *Zea mays* [19]. Mature leaves are generally less sensitive to drought stress compared to juvenile leaves [20]. Furthermore, mature leaves are more developed and have the ability to better respond to plant diseases, insect pests, nutritional stress and etc. [21]. However, little research has thus far been conducted on the application of proteomics to the mature organs (especially leaves) of perennial plants. A simple, economical, and reliable method for protein sample preparation from various plants has not yet been established. The work presents a sample preparation method for two-dimensional gel electrophoresis of mature, recalcitrant leaves of perennial plants using the leaves of six common perennial plants (including herbs, vines, and woody plants) to provide important technological support for proteomics studies in perennial plants.

## Materials and Methods

### Experiment materials

**Figure 1:**
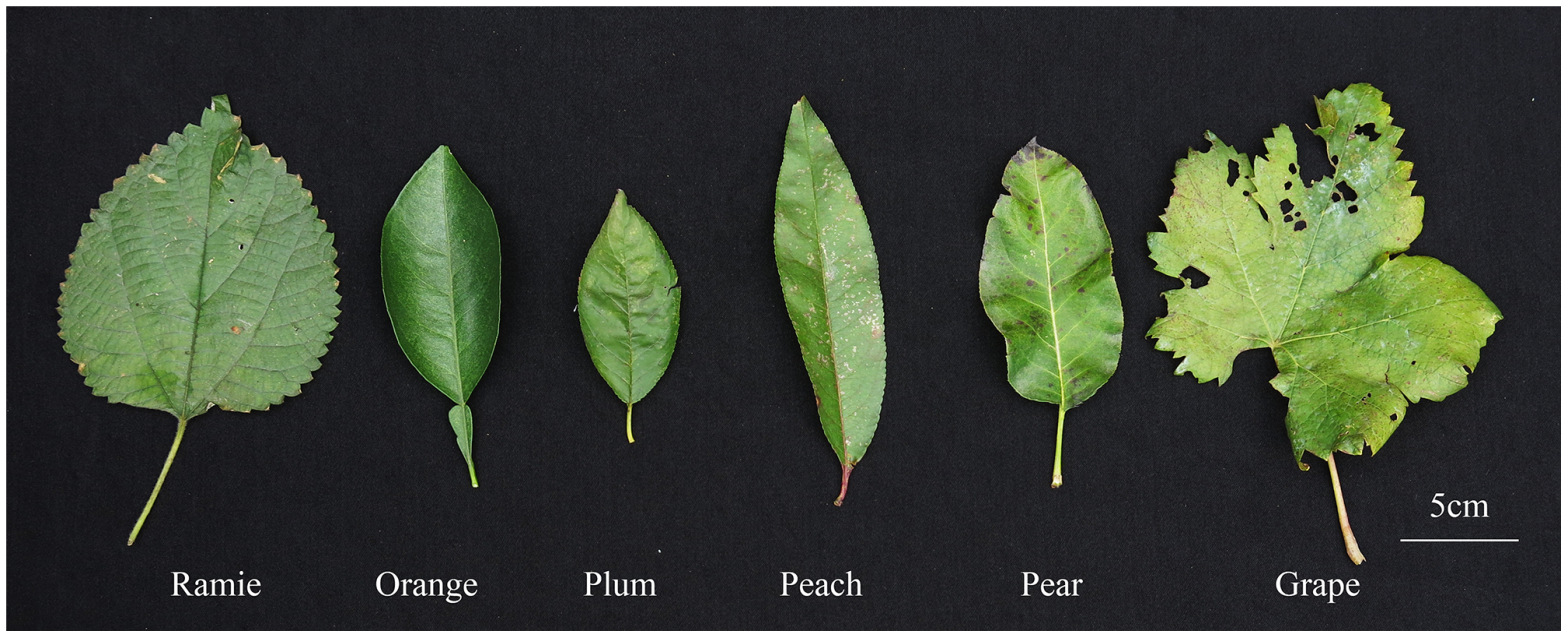
Mature leaves of six perennial plants.

The experiment materials consisted of the mature leaves of six perennial plants (from the experiment base of Huazhong Agricultural University) (See Fig. 1):

Herbs: ramie (*Boehmeria nivea* L.Gaud.), cultivar “Huazhu No. 5”, 5 year old; vine: grape (*Vitis vinifera*), cultivar “Red Fuji menglisha”, 7 year old; woody plants: pear (*Pyrus* spp.), cultivar “Fengshui”, 8 year old; plum (*Prunus salicina* Lindl.), cultivar “Heihupo”, 3 year old; peach (*Prunus persica* L.), cultivar “Annongshuimi”, 8 year old; orange (*Citrus sinensis* L. Osbeck), cultivar “Newhall”,7 year old.

For each species, 2 g of mature leaf material was used. This sampling was repeated a total of three times from three different individuals. The leaf samples were immediately stored in liquid nitrogen and preserved in a freezer at − 80 °C prior to protein exaction.

### Sample preparation

The protein samples were extracted using the TCA/acetone method reported by Deng *et al*. (2013) [22]. Samples of 1 g from the leaves of the different species were ground completely into powder in liquid nitrogen for cell disruption, and 10 mL of extraction solution consisting of 10% TCA and 0.07% *β*-mercaptoethanol in cold acetone (−20°C) containing 1 mmol/L PMSF (4°C) was added. The sample was incubated overnight at −20°C. Centrifugation was then conducted at 12842 *g* at 4°C for 30 min, and the supernatant was discarded. After precipitation, 10 mL of 80% cold acetone (−20°C) containing 1 mmol/L PMSF (4°C) was immediately added. The sample was kept at −20°C for 1 h. Centrifugation was then conducted at 12842 *g* at 4°C for 30 min, and the supernatant was discarded. This procedure was repeated three times in total. The precipitate was dried under vacuum and dried pellet weighed.

Subsequent protein extraction and purification was conducted using two methods. The first, reported by Deng *et al*. (2013) [22], was a protein extraction and purification technique previously established for tender roots, leaves and stems (Method A), the method was as follows. The protein lysis buffer 1 (7 M urea, 2 M thiourea, 4% CHAPS, 1% DTT) was added to the dry protein (0.1 g) powder at 15 µL/mg; kept at room temperature for 2 h; then centrifuged at 30000 *g* for 30 min at 24°C, and the precipitate was discarded; four volumes of 100% acetone (−20°C) was then added to the supernatant for 1.5 h. After centrifuged at 12842 *g* for 15 min at 4°C, the supernatant was discarded; the precipitate was dried under vacuum. After that, about 400 µL protein lysis buffer 1 was added to dissolve the precipitate (protein). For the second method (Method B), the procedure was as follows. The protein lysis buffer 2 (5 M urea, 2 M thiourea, 1% Triton X-100, 50 mM Tris-HCl (pH 8.8), 1% DTT) was added to the dry protein pellet (0.1 g) at a ratio of 15 µL/mg. The sample was left at room temperature for 2 h, then placed in an ultrasonic water bath at 25–30°C for 15 min. Centrifugation was then performed at 30,000 *g* at 24°C for 30 min, and the supernatant was retained. After centrifugation, a volume of 20% TCA-water solution (4°C) equal to five times the volume of the supernatant was added. The sample was placed on ice to incubate for 10 min. Centrifugation was then performed at 12842 *g* at 4°C for 15 min, and the supernatant was discarded. A 1.5 mL volume of 100% acetone (−20°C) was then added to the precipitate. After mixing completely, the sample was placed at −20°C for 1.5 h. Centrifugation was performed at 12842 *g* at 4°C for 15 min, and the supernatant was discarded. The precipitate was dried under vacuum. Subsequently, about 400 µL volume of lysis buffer 3 without Tris (5 M urea, 2 M thiourea, 1% Triton X-100, 1% DTT) was added to dissolve the precipitate and obtain the protein sample. The protein concentration was measured by the Bradford assay [23]. These procedures were independently repeated a total of three times.

### Two-dimensional gel electrophoresis

The protein solution was added at a ratio of 150 µg/IPG strip (Bio-rad). Lysis buffer 1 was added to make a total volume of 300 µL. Centrifugation was then performed at 30,000 *g* at 24°C for 30 min, and the supernatant was obtained for IPG strip rehydration. The rehydration was passive hydration, and the duration was 12–14 h at room temperature. After the rehydration, isoelectric focusing was carried out on a Protean IEF Cell (Bio-rad) with the following settings: S1 250 V 10 min, S2 500 V 30 min, S3 1000 V 1 h, S4 9000 V 5 h, S5 50000 VH (ramie sample)/55000 VH (other 5 plant samples), S6 500 V 1 h. After focusing, the strips were put into 5 mL of equilibrium buffer (6 M Urea, 2% SDS, 0.375 M, pH 8.8, Tris-HCl, 20% Glycerol); 0.05 g of DTT was added with gentle shaking on a shaker for 15 min in order to reach the first equilibrium; the strips were placed in the equilibrium buffer again; 0.255 g of IAA was added with gentle shaking for 15 min to reach the second equilibrium. A 12% polyacrylamide gel was used for the second dimension electrophoresis, the step was performed at 18°C in PROTEAN II XI (Bio-rad) with the following program: 10 mA, 1 h; 30 mA, 3.5 h. The SDS-PAGE two-dimensional electrophoresis of samples was undertaken a total of two times from two independent extractions.

### Silver-staining and photography

The gels were placed in the fixative liquid (40% v/v Ethanol, 10% v/v acetic acid, 50% v/v deionized water) for a fixation period of 3 h. After fixation, the fixative liquid was discarded, and the sensitizing solution was added (2 g/L sodium thiosulfate, 34 g/L sodium acetate) for a sensitization period of 30 min. After the sensitization step, deionized water was used to wash the gel three times, with each wash lasting for 5 min. The silver-staining solution (2.5 g/L silver nitrate, 0.02% v/v formaldehyde) was then added for a dark staining period of 20 min. After staining, deionized water was used to wash the gel twice, with each wash lasting for 30 s. Next, a developer solution (25 g/L sodium carbonate, 0.04% v/v formaldehyde) was added for color development period of 3–5 min. After color development, the stop solution (15 g/L EDTA.Na_2_) was added. Finally, the gel was scanned using a GS-800 (Bio-Rad).

#### Analytical method

All gels were imaged using a GS-800 (Bio-Rad), and all images were done with filter wizard and protein spots number detected by the PDQuest 8.01 software (Bio-Rad). SAS 9.0 [24] was used for statistical analysis. Differences in protein concentration between method A and B were analyzed using a *t* test (*n* = 3).

## Results and Discussion

### Protein concentration

The data outlined in Table 1 indicate that the two sample preparation methods used for each of the six plants both obtained a relatively high protein concentration. The protein concentration obtained by Method B was significantly higher than that obtained by Method A in plum, peach, pear, and orange; for grape, the protein concentration for Method A was significantly higher than that for Method B, but Method A and B both obtained a relatively high protein concentration (8.65 mg/mL and 7.80 mg/mL). For ramie samples, the difference between the two methods was not significant. Therefore, among the plant species tested here, Method B is superior over Method A in most cases, even though Method A should not be disregarded. The superiority of Method B may have been due to the addition of 50 mM Tris-HCl (pH 8.8), which provided an alkaline environment and increased the ion concentration. This environment is beneficial for protein dissolution [25]. In addition, Triton X-100 is cheaper than CHAPS, which reduces the cost of protein sample preparation.

**Table 1:** Protein concentrations obtained from two protein sample preparation methods (µg/µL).

Method	Grape	Plum	Peach	Pear	Orange	Ramie
Method A	8.65±0.13*	1.57±0.07	3.48±0.22	1.81±0.06	3.11±0.08	5.40±0.14
Method B	7.80±0.12	3.54 ±0.05*	7.50±0.08*	2.17±0.13*	4.83±0.09*	5.19±0.07

In the table, the value is expressed as mean±SD, *n* = 3;

*p = 0.05 indicates a significant difference; the sampling amount of all six plants was 0.1 g; the total volume of the samples were 400 µL.

### Two-dimensional gel electrophoresis analysis

**Figure 2:**
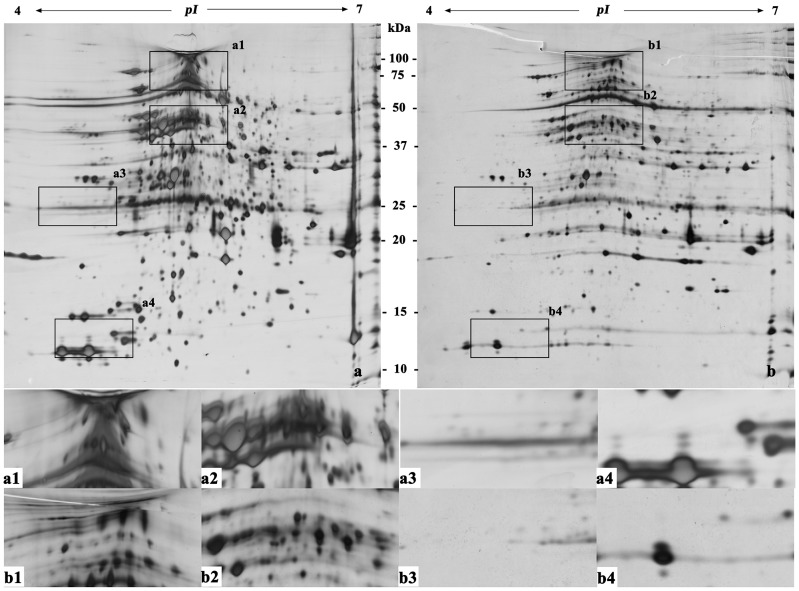
Two-dimensional electrophoretogram of proteins from mature leaves of grape. The sample loading amount was 150 µg/strip; IPG strip used was pH 4–7, 17 cm, linear; 12% polyacrylamide gel. a. Method a; b. Method B. a1–a4, b1–b4 show the different areas between gel a and gel b.

**Figure 3:**
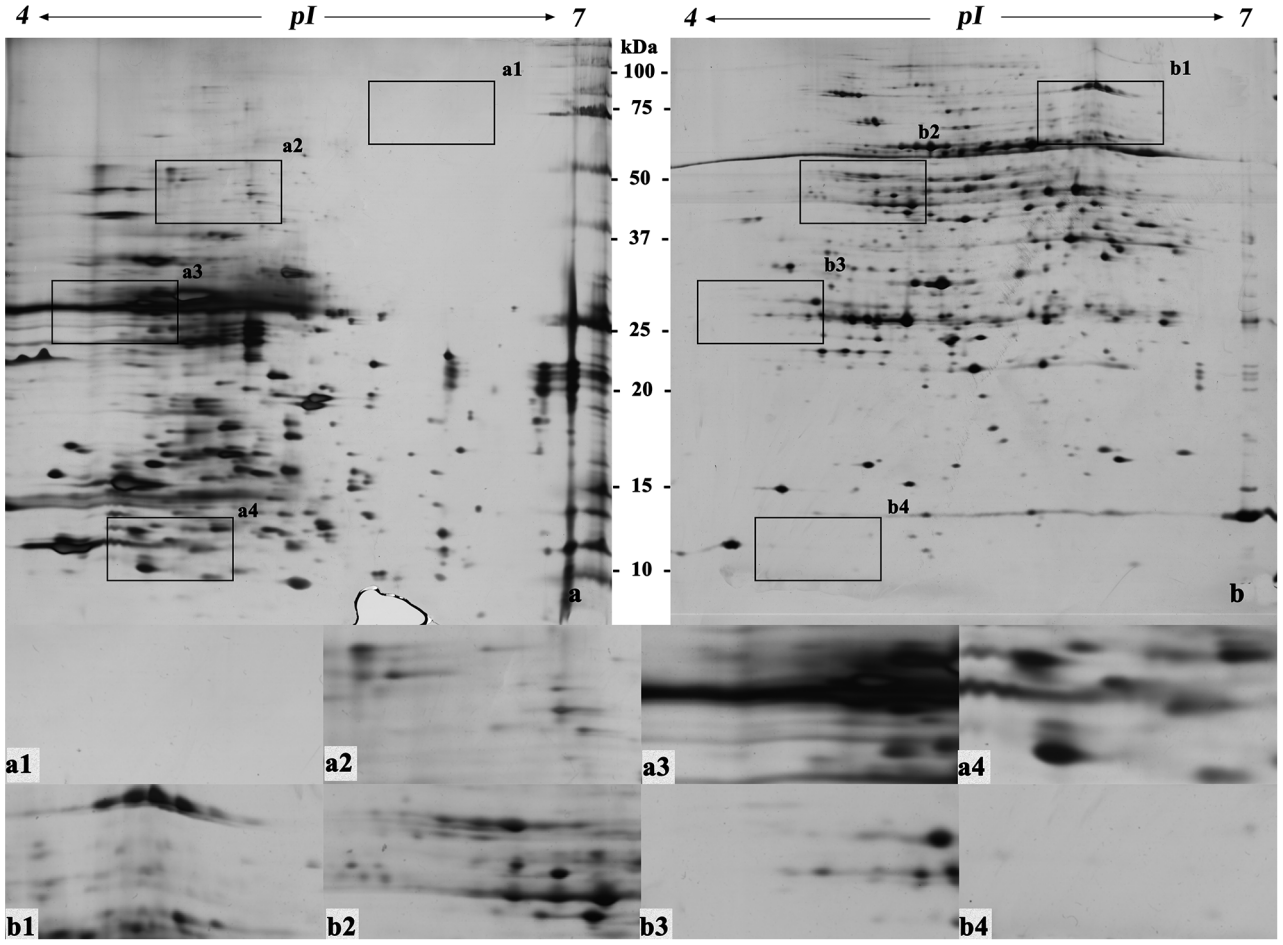
Two-dimensional electrophoretogram of proteins from mature leaves of plum. The sample loading amount was 150 µg/strip; IPG strip used was pH 4–7, 17 cm, linear; 12% polyacrylamide gel. a. Method A; b. Method B. a1–a4, b1–b4 show the different areas between gel a and gel b.

**Figure 4:**
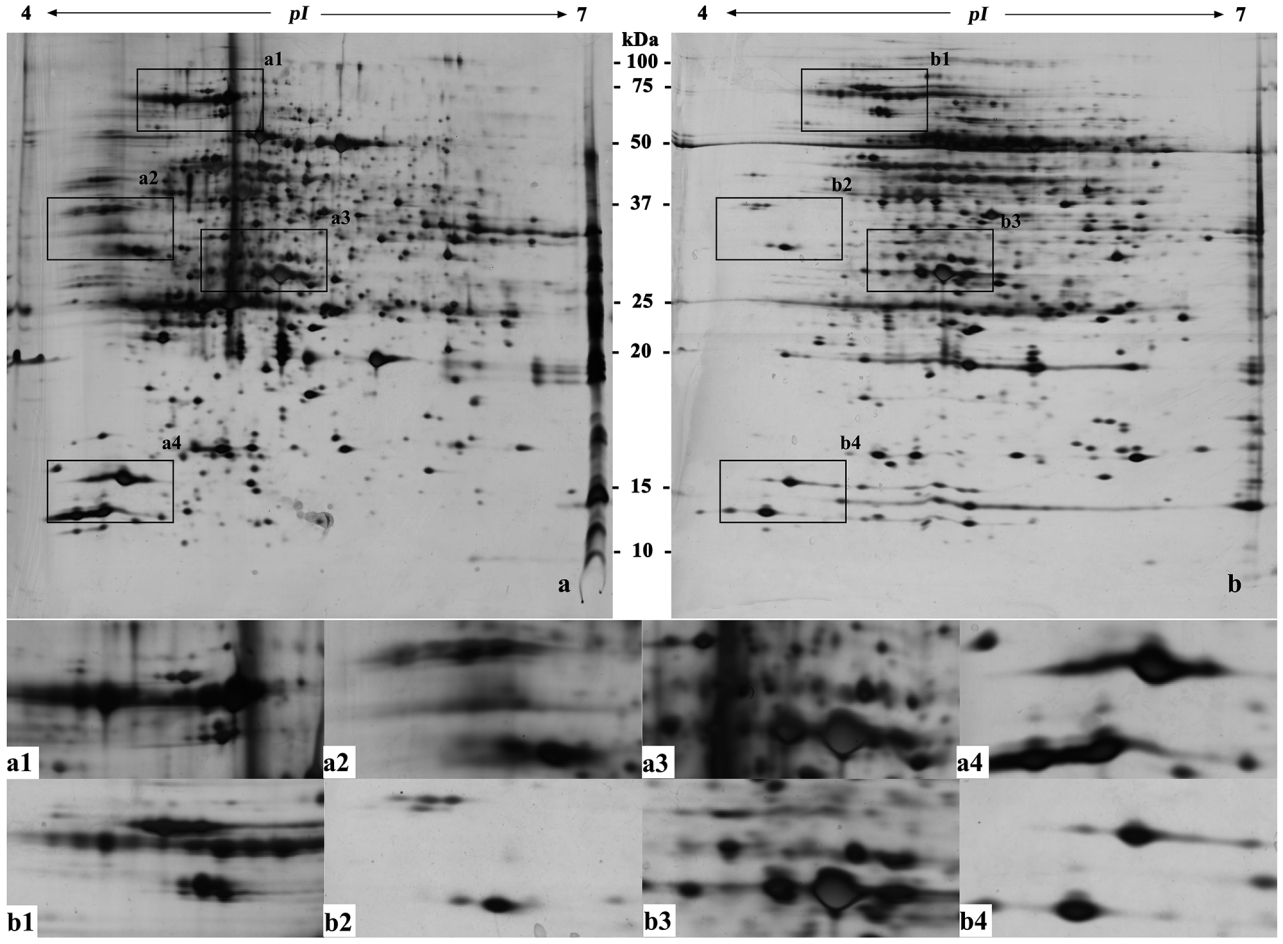
Two-dimensional electrophoretogram of proteins from mature leaves of peach. The sample loading amount was 150 µg/strip; IPG strip used was pH 4–7, 17 cm, linear; 12% polyacrylamide gel. a. Method A; b. Method B. a1–a4, b1–b4 show the different areas between gel a and gel b.

**Figure 5:**
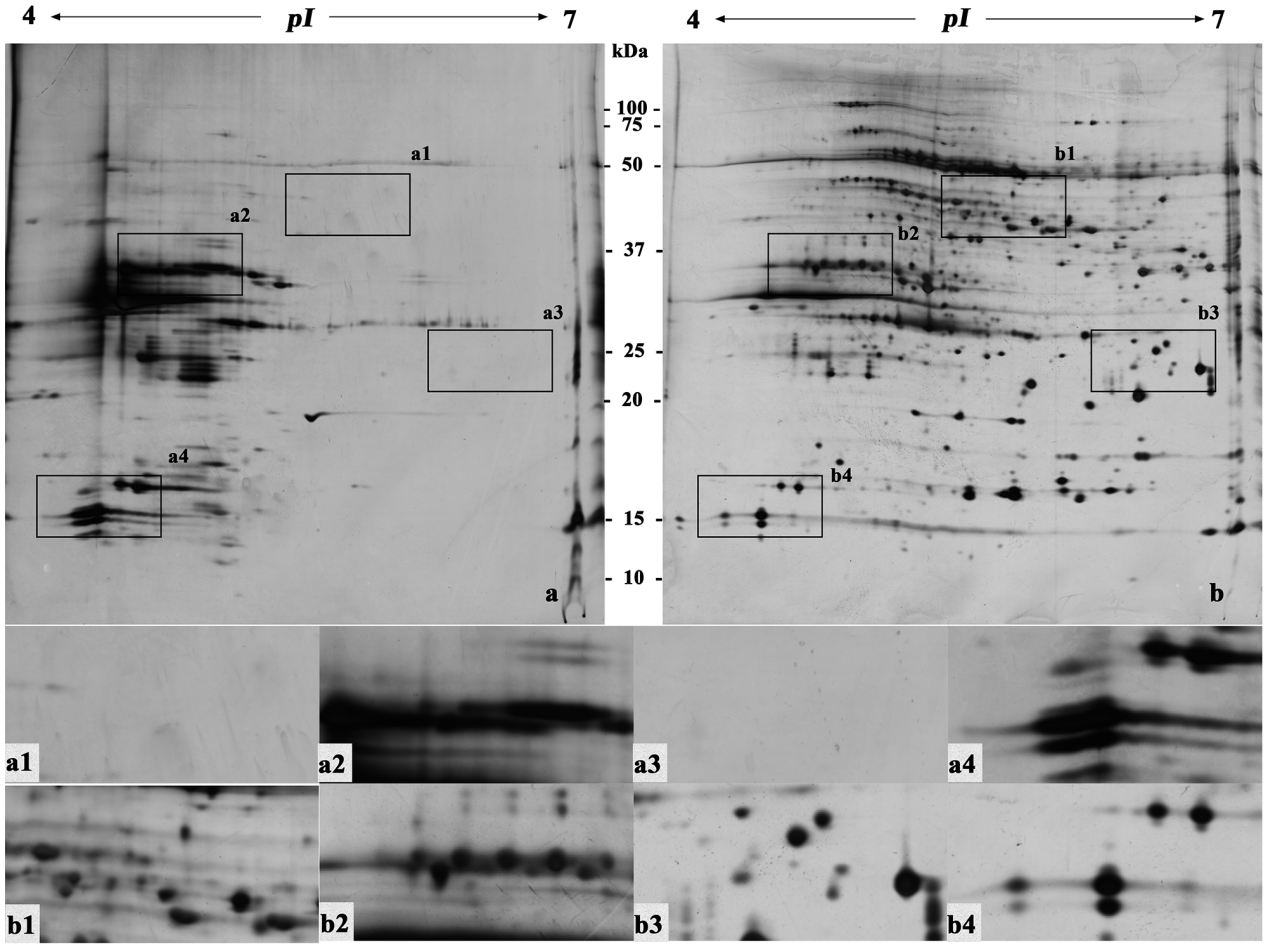
Two-dimensional electrophoretogram of proteins from mature leaves of pear. The sample loading amount was 150 µg/strip; IPG strip used was pH 4–7, 17 cm, linear; 12% polyacrylamide gel. a. Method A; b. Method B. a1–a4, b1–b4 show the different areas between gel a and gel b.

**Figure 6:**
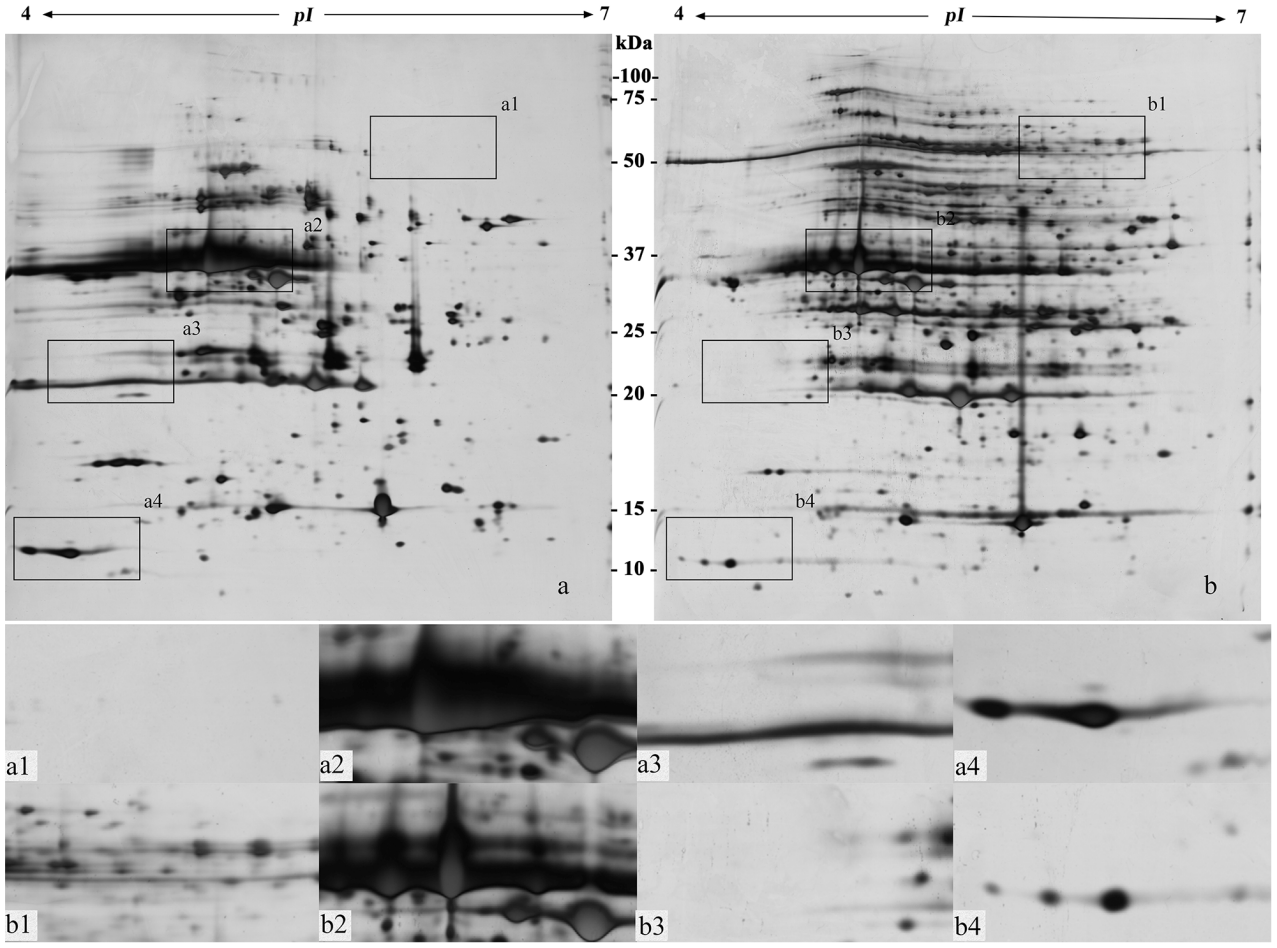
Two-dimensional electrophoretogram of proteins from mature leaves of orange. The sample loading amount was 150 µg/strip; IPG strip used was pH 4–7, 17 cm, linear; 12% polyacrylamide gel. a. Method A; b. Method B. a1–a4, b1–b4 show the different areas between gel a and gel b.

**Figure 7:**
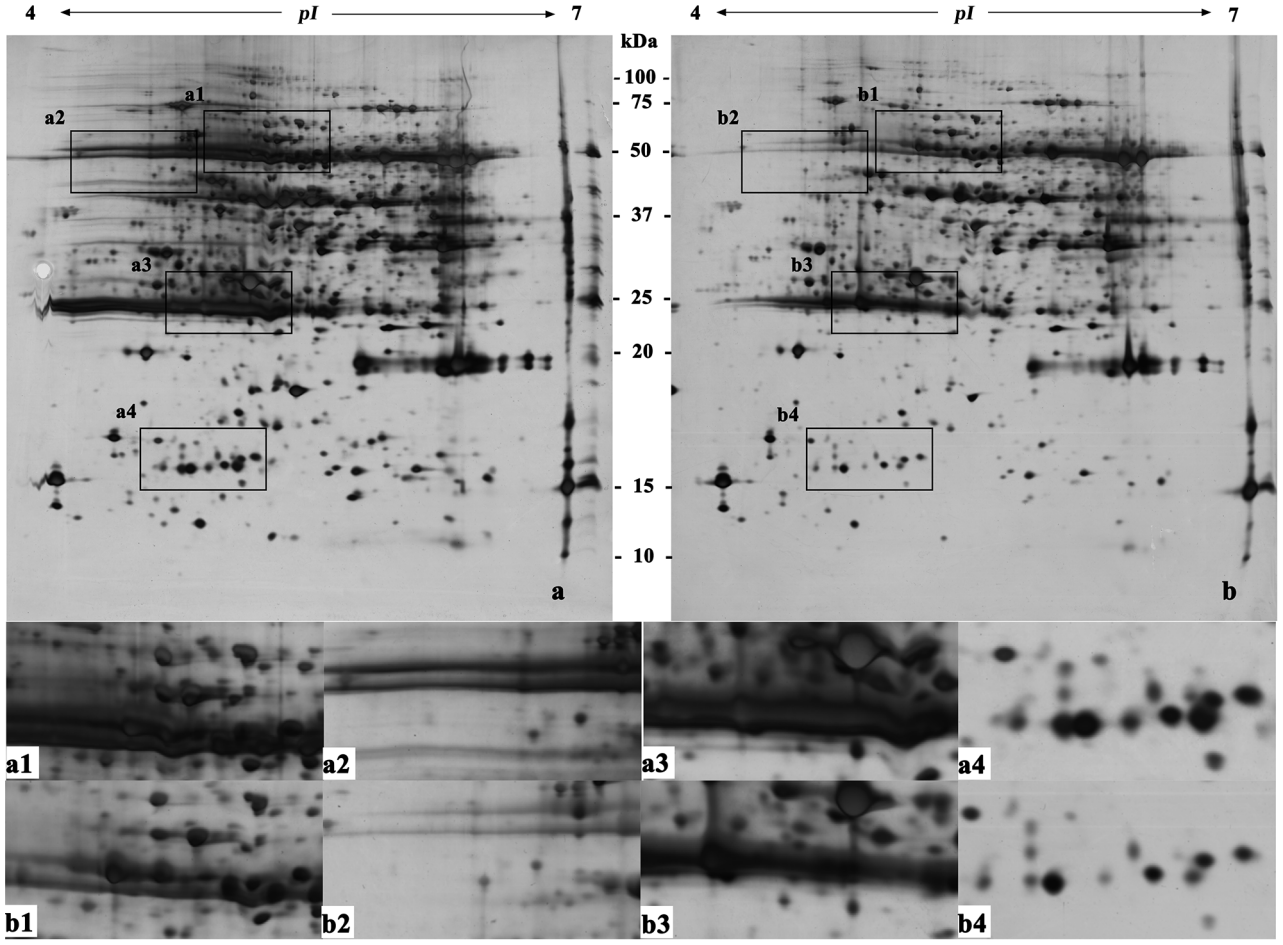
Two-dimensional electrophoretogram of proteins from mature leaves of ramie. sample loading amount was 150 µg/strip; IPG strip used was pH 4–7, 17 cm, linear; 12% polyacrylamide gel. a. Method A; b. Method B. a1–a4, b1–b4 show the different areas between gel a and gel b.

Protein extraction and preparation is the basis of and one of the key processes in two-dimensional gel electrophoresis [4]. Figs. 2–7 (Figs. S1, S2, S3, S4, S5 and S6) show that the two-dimensional gel electrophoresis maps of Method B were clearer than those of Method A, with the nonprotein substances that can result in poor focusing essentially removed, especially for plum, pear and orange. Method A was able to discriminate between a large quantity of protein spots for peach and ramie, but the gel background was darker and more stained, as seen in Figs. 4-a1 and a2 and Fig. 7-a1. Moreover, the protein spots were fewer for plum, pear and orange (Figs. 3, 5, and 6). The proteins and impurities were concentrated within a certain area, as seen in Fig. 5-a2, Fig. 3, and Fig. 6-a3. In addition, Fig. 2, Fig. 3, Fig. 6-a3, Fig. 5-a4, Fig. 4, and Fig. 7-a2 indicate that the protein gels of the six plants still contained large amounts of horizontal streaking. Therefore, Method A was unable to eliminate the gel background noise caused by nucleic acids [26], as well as the horizontal streaking caused by polysaccharides and phenols. The polysaccharides likely caused the aggregation of the protein samples, which blocked the pores of the SDS-PAGE gel, preventing the proteins from passing through and focusing them in a certain area [2]. As the mature, recalcitrant leaves of perennial plants contain large amounts of cellulose, pectin and nonprotein substances such as polysaccharides, lipids, and organic acids [6], [27]. These substances cannot be easily removed by common preparation methods (including Method A) and this may be the reason that there is little comparative proteome analysis on mature, recalcitrant leaves of perennial plants. In comparison, however, Method B appears to have eliminated the impurities, which indicates Method B would be more suitable for mature, recalcitrant leaves of perennial plants.

To better compare the resolved proteins using the two extraction techniques, we examined the number of proteins that could confidently be identified for each 2-DE gel when analyzed by the PDQuest 8.01 software (Table 2). The total number of proteins detected was considerably higher for plant samples extracted using Method B for all plant varieties, supporting the qualitative analysis of these results. Compared with Method A, the number of proteins increased from 2.12% to 380.2% when using Method B. These increases were more than 100% in plum and pear for the first sample and were more than 20% for the second sample. However, the increase was less than 20% in peach and ramie for both samples analyzed. (Table 2).

**Table 2:** Spot numbers detected in the two protein sample preparation methods.

Method	Grape		Plum		Peach		Pear		Orange		Ramie	
	FS	SS	FS	SS	FS	SS	FS	SS	FS	SS	FS	SS
Method A	586	856	224	702	614	750	111	246	351	631	698	800
Method B	655	1065	541	853	627	838	533	422	598	786	794	854
IR(%)	11.78	24.42	141.5	21.51	2.12	11.73	380.2	71.54	70.37	24.56	13.75	6.75

FS: the first sample; SS: the second sample. IR: increment rate, IR (%)  =  [(A − B)/A] ×100.

In addition, this research also found that the high-abundance proteins resolved better when extracted using Method B than Method A, which can assist in the identification of low-abundance proteins that are often masked by poorly focused high-abundance proteins (e.g., rubisco) [28], as seen in Figs. 2-b1 and b2 and Fig.2–Fig.7-b4. In addition, Method B greatly enhanced the resolution of high-molecular-weight proteins in some species in comparison with Method A, as seen in Fig.2–Fig.4-b1. This phenomenon is similar to the result reported by Molloy *et al*. (1998) [29], who showed that Tris was helpful for the separation and identification of high-molecular-weight membrane proteins. However, Method B showed an absence of certain proteins in the low-molecular-weight area 10–15 kDa of pH 4-5, as seen in Fig. 2–Fig. 7-b4. This result may have been due to the addition of a strong acid, 20% TCA (pH<4) during the sample preparation, which may have caused acid-mediated protein hydrolysis [30].

## Conclusion

This paper established a sample preparation system suitable for the mature, recalcitrant leaves of perennial plants. The lysis buffer was 5 M urea, 2 M thiourea, 1% Triton X-100, 50 mM Tris-HCl (pH 8.8), and 1% DTT. After protein extraction, a volume of 20% TCA-water solution equal to five times the volume of the supernatant and 100% precooled acetone were added for the purification of the protein extract. This method is suitable for sample preparation of mature recalcitrant leaves of perennial plants, including ramie (herb), grape (vine), plum, pear, peach, and orange (woody plants). The method enhances the resolution of both high-molecular-weight proteins and low-abundance proteins. This sample preparation method provides a simple, widely applicable and economically feasible technological tool for the proteomic study of mature recalcitrant leaves of perennial plants.

## Supporting Information

Figure S1 **Two-dimensional electrophoretogram of proteins from mature leaves of grape.** The sample loading amount was 150 µg/strip; IPG strip used was pH 4–7, 17 cm, linear; 12% polyacrylamide gel. a. Method A; b. Method B.
(TIF)

Figure S2 **Two-dimensional electrophoretogram of proteins from mature leaves of plum.** The sample loading amount was 150 µg/strip; IPG strip used was pH 4–7, 17 cm, linear; 12% polyacrylamide gel. a. Method A; b. Method B.
(TIF)

Figure S3 **Two-dimensional electrophoretogram of proteins from mature leaves of peach.** The sample loading amount was 150 µg/strip; IPG strip used was pH 4–7, 17 cm, linear; 12% polyacrylamide gel. a. Method A; b. Method B.
(TIF)

Figure S4 **Two-dimensional electrophoretogram of proteins from mature leaves of pear.** The sample loading amount was 150 µg/strip; IPG strip used was pH 4–7, 17 cm, linear; 12% polyacrylamide gel. a. Method A; b. Method B.
(TIF)

Figure S5 **Two-dimensional electrophoretogram of proteins from mature leaves of orange.** The sample loading amount was 150 µg/strip; IPG strip used was pH 4–7, 17 cm, linear; 12% polyacrylamide gel. a. Method A; b. Method B.
(TIF)

Figure S6 **Two-dimensional electrophoretogram of proteins from mature leaves of ramie.** sample loading amount was 150 µg/strip; IPG strip used was pH 4–7, 17 cm, linear; 12% polyacrylamide gel. a. Method A; b. Method B.
(TIF)

## References

[1] Wasinger VC, Cordwell SJ, Cerpa PA, Yan JX, Gooley AA, et al (1995) Progress with gene-product mapping of the mollicutes: *Mycoplasma genitalium* . Electrophoresis 16: 1090–1094. 7498152 10.1002/elps.11501601185

[2] Bodzon-Kulakowska A, Bierczynska-Krzysik A, Dylag T, Drabik A, Suder P, et al (2007) Methods for samples preparation in proteomic research. J Chromatogr B 849: 1–31. 10.1016/j.jchromb.2006.10.040 17113834

[3] Görg A, Weiss W, Dunn MJ (2004) Current two-dimensional electrophoresis technology for proteomics. Proteomics 4: 3665–3685. 15543535 10.1002/pmic.200401031

[4] Isaacson T, Damasceno CMB, Saravanan RS, He Y, Catala C, et al (2006) Sample extraction techniques for enhanced proteomic analysis of plant tissues. Nat Protocols 1: 769–774. 17406306 10.1038/nprot.2006.102

[5] Freeman WM, Hemby SE (2004) Proteomics for protein expression profiling in neuroscience. Neurochem Res 29: 1065–1081. 15176464 10.1023/b:nere.0000023594.21352.17 PMC3843356

[6] Wang W, Scali M, Vignani R, Spadafora A, Sensi E, et al (2003) Protein extraction for two- dimensional electrophoresis from olive leaf, a plant tissue containing high levels of interfering compounds. Electrophoresis 24: 2369–2375. 12874872 10.1002/elps.200305500

[7] Rose JKC, Bashir S, James JG, Jahn MM, Saravanan RS (2004) Tackling the plant proteome: practical approaches, hurdles and experimental tools. Plant J 39: 715–733. 15315634 10.1111/j.1365-313X.2004.02182.x

[8] Saravanan RS, Rose JK (2004) A critical evaluation of sample extraction techniques for enhanced proteomic analysis of recalcitrant plant tissues. Proteomics 4: 2522–2532. 15352226 10.1002/pmic.200300789

[9] Vâlcu CM, Schlink K (2006) Efficient extraction of proteins from woody plant samples for two-dimensional electrophoresis. Proteomics 6: 1599–1605. 16791823 10.1002/pmic.200500660

[10] Dai S, Li L, Chen T, Chong K, Xue Y, Wang T (2006) Proteomic analyses of *Oryza sativa* mature pollen reveal novel proteins associated with pollen germination and tube growth. Proteomics 6: 2504–2529. 16548068 10.1002/pmic.200401351

[11] Magni C, Scarafoni A, Herndl A, Sessa F, Prinsi B, et al (2007) Combined 2D electrophoretic approaches for the study of white lupin mature seed storage proteome. Phytochemistry 68: 997–1007. 17320919 10.1016/j.phytochem.2007.01.003

[12] Rashid A, Badhan A, Deyholos M, Kav N (2013) Proteomic Profiling of the Aleurone Layer of Mature *Arabidopsis thaliana* Seed. Plant Mol Biol Rep 31: 464–469.

[13] Kottapalli KR, Payton P, Rakwal R, Agrawal GK, Shibato J, et al (2008) Proteomics analysis of mature seed of four peanut cultivars using two-dimensional gel electrophoresis reveals distinct differential expression of storage, anti-nutritional, and allergenic proteins. Plant Sci 175: 321–329.

[14] Bancel E, Rogniaux H, Debiton C, Chambon C, Branlard G (2010) Extraction and proteome analysis of starch granule-associated proteins in mature wheat kernel (*Triticum aestivum* L.). J Proteome Res 9: 3299–3310. 20481496 10.1021/pr9010525

[15] Meziani S, Nadaud I, Gaillard-Martinie B, Chambon C, Benali M, et al (2012) Proteomic analysis of the mature kernel aleurone layer in common and durum wheat. J Cereal Sci 55: 323–330.

[16] Wu QF, Li C, Ke LM, Jiao CJ, Jiang JL, et al (2011) A high-efficiency, two-dimensional gel electrophoresis platform for mature leaves of grass pea (*Lathyrus sativus* L.). Acta Physiol Plant 33: 2387–2397.

[17] Islam N, Lonsdale M, Upadhyaya NM, Higgins TJ, Hirano H, et al (2004) Protein extraction from mature rice leaves for two-dimensional gel electrophoresis and its application in proteome analysis. Proteomics 4: 1903–1908. 15221747 10.1002/pmic.200300816

[18] Noir S, Bräutigam A, Colby T, Schmidt J, Panstruga R (2005) A reference map of the *Arabidopsis thaliana* mature pollen proteome. Biochem Bioph Res Co 337: 1257–1266. 10.1016/j.bbrc.2005.09.185 16242667

[19] Zhu Y, Zhao P, Wu X, Wang W, Scali M, et al (2011) Proteomic identification of differentially expressed proteins in mature and germinated maize pollen. Acta Physiol Plant 33: 1467–1474.

[20] Taulavuori E, Tahkokorpi M. Laine K, Taulavuori K (2010) Drought tolerance of juvenile and mature leaves of a deciduous dwarf shrub *Vaccinium myrtillus* L. in a boreal environment. Protoplasma 241: 19–27. 20169457 10.1007/s00709-009-0096-x

[21] Wong M (2006) Mature leaf chlorosis and necrosis. Landscape 50: 15.

[22] Deng G, Liu L, Wang H, Lao C, Wang B, et al (2013) Establishment and Optimization of Two-dimensional Electrophoresis (2-DE) Technology for Proteomic Analysis of Ramie. Int J Agric Biol 15: 570–574.

[23] Bradford MM (1976) A rapid and sensitive method for the quantization of microgram quantities of protein utilizing the principle of protein-dye binding. Anal Biochem 72: 248–254. 942051 10.1016/0003-2697(76)90527-3

[24] SAS Institute Inc., 1989. SAS/STAT users guide, Version 6, Fourth edition. SAS Institute Inc., Cary.

[25] Gelfi C, Righetti PG (1983) Preparative isoelectric focusing in immobilized pH gradients. II. A case report. J biochem Biop Meth 8: 157–172. 10.1016/0165-022x(83)90041-6 6643921

[26] Leimgruber RM, Malone JP, Radabaugh MR, LaPorte ML, Violand BN, et al (2002) Development of improved cell lysis, solubilization and imaging approaches for proteomic analyses. Proteomics 2: 135–144. 11840559

[27] Aerts R, Van der Peijl M J (1993) A simple model to explain the dominance of low-productive perennials in nutrient-poor habitats. Oikos 66: 144–147.

[28] Wang W, Tai FJ, Chen SN (2008) Optimizing protein extraction from plant tissues for enhanced proteomics analysis. J Sep Sci 31: 2032–2039. 18615819 10.1002/jssc.200800087

[29] Molloy MP, Herbert BR, Walsh BJ, Tyler MI, Traini M, et al (1998) Extraction of membrane proteins by differential solubilization for separation using two-dimensional gel electrophoresis. Electrophoresis 19: 837–844. 9629924 10.1002/elps.1150190539

[30] Zellner M, Winkler W, Hayden H, Diestinger M, Eliasen M, et al (2005) Quantitative validation of different protein precipitation methods in proteome analysis of blood platelets. Electrophoresis 26: 2481–2489. 15895463 10.1002/elps.200410262

