# Supplementary figures and images for: A Proteomics Sample Preparation Method for Mature, Recalcitrant Leaves of Perennial Plants
Source: PLoS One. 2014 Jul 16;9(7):e102175. doi: 10.1371/journal.pone.0102175 (PMC4100801; doi:10.1371/journal.pone.0102175)

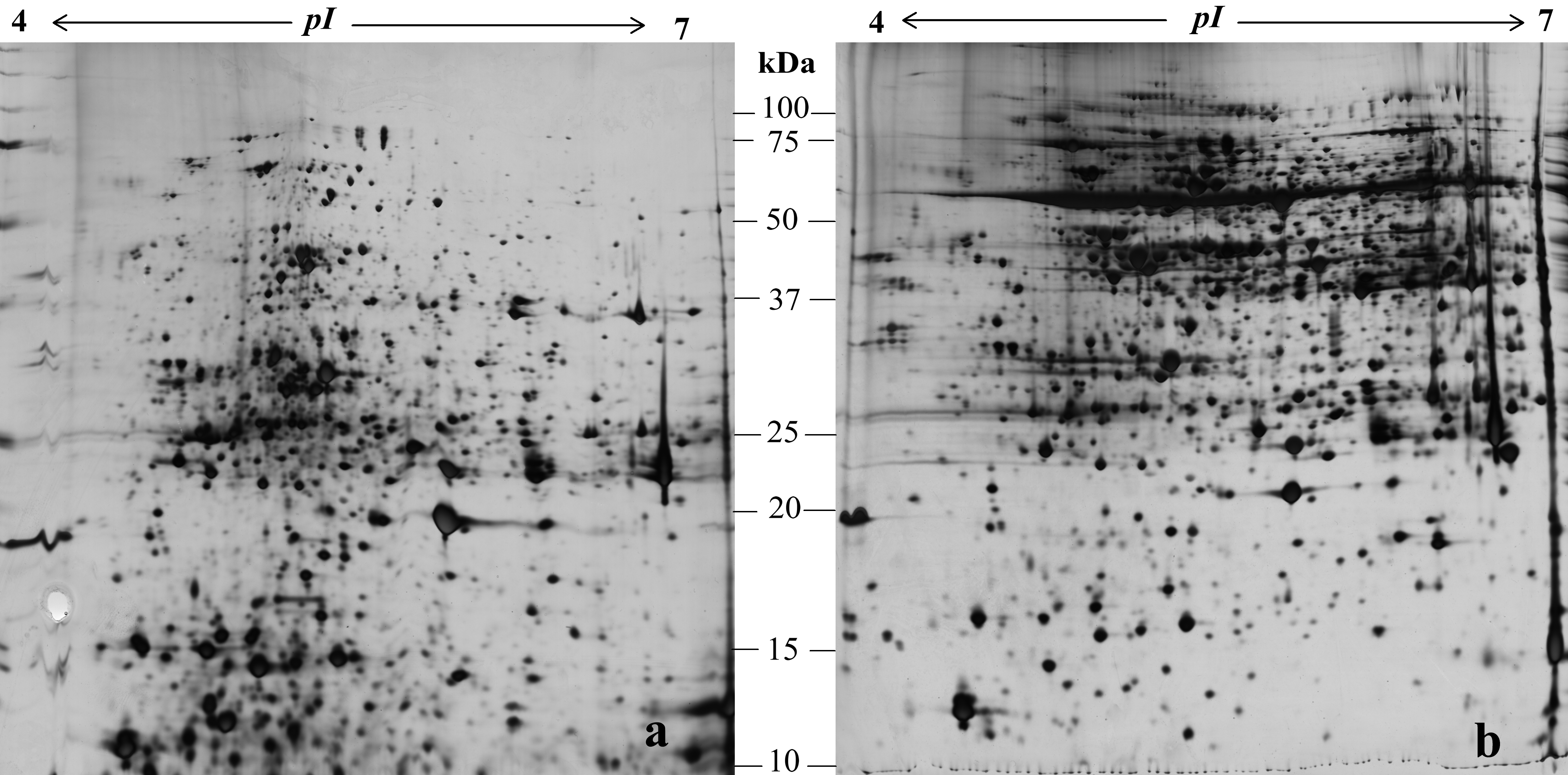

Supplement: Figure S1 — Two-dimensional electrophoretogram of proteins from mature leaves of grape. The sample loading amount was 150 µg/strip; IPG strip used was pH 4–7, 17 cm, linear; 12% polyacrylamide gel. a. Method A; b. Method B. (TIF) [file pone.0102175.s001.tif]

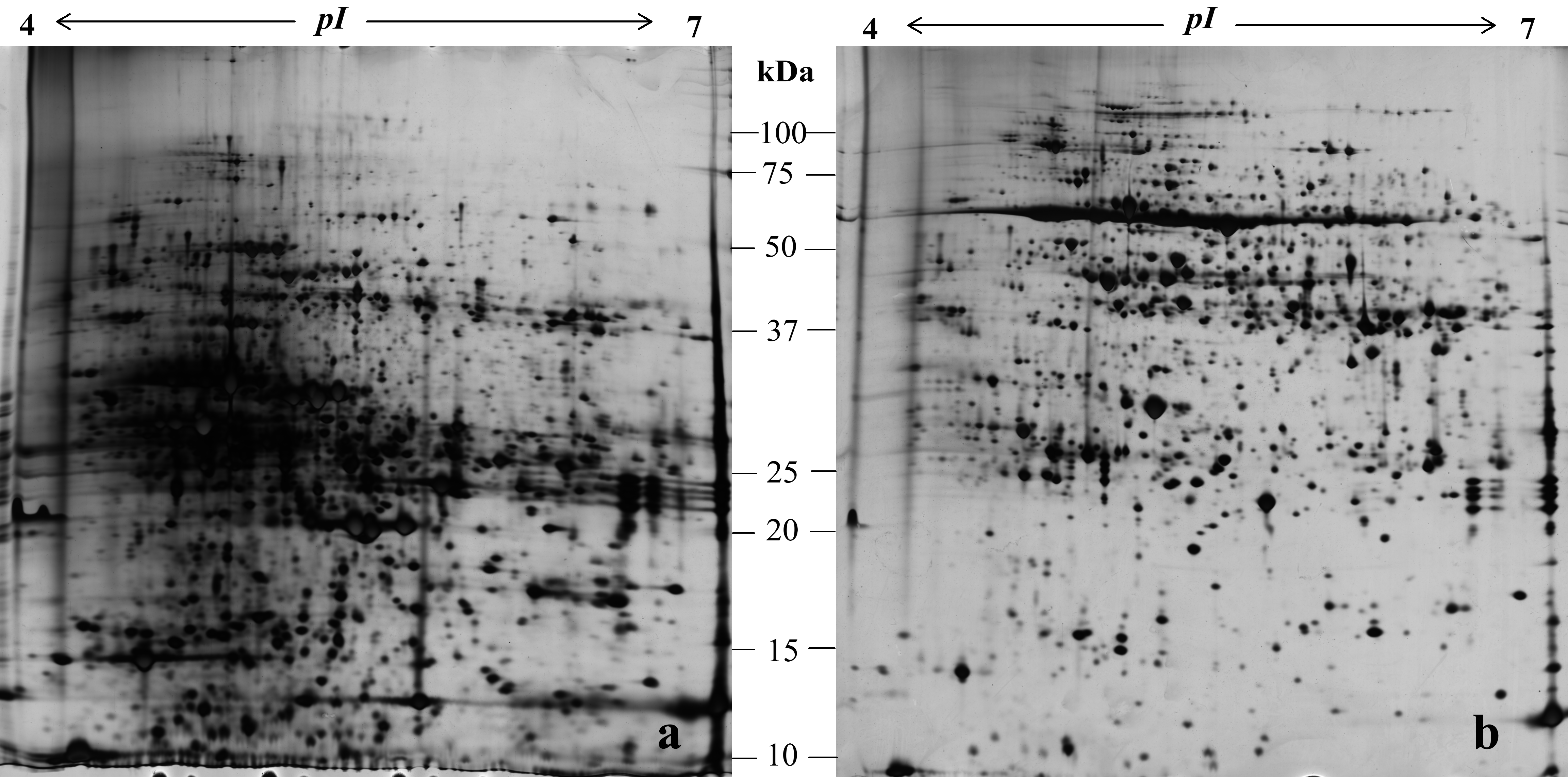

Supplement: Figure S2 — Two-dimensional electrophoretogram of proteins from mature leaves of plum. The sample loading amount was 150 µg/strip; IPG strip used was pH 4–7, 17 cm, linear; 12% polyacrylamide gel. a. Method A; b. Method B. (TIF) [file pone.0102175.s002.tif]

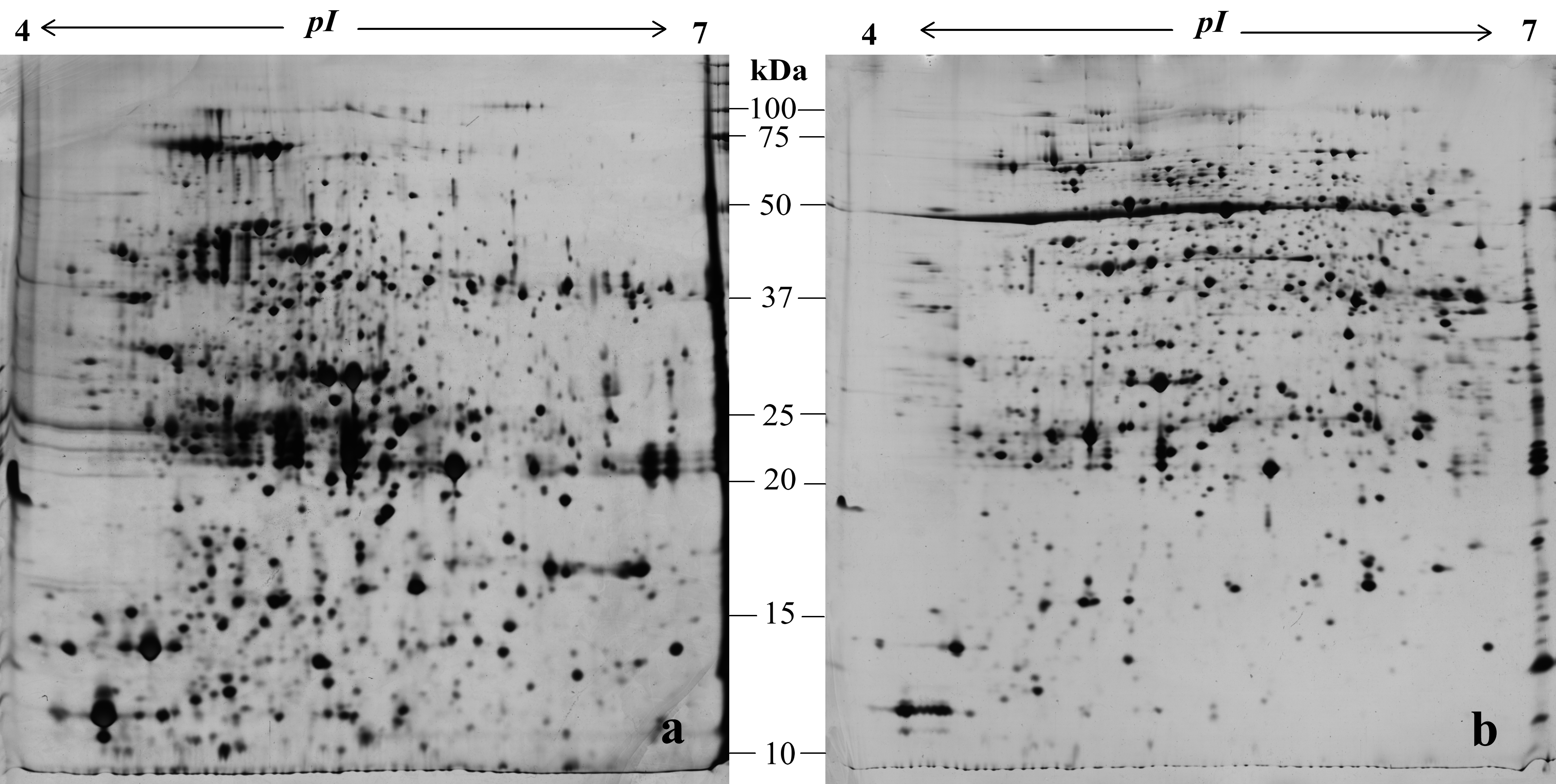

Supplement: Figure S3 — Two-dimensional electrophoretogram of proteins from mature leaves of peach. The sample loading amount was 150 µg/strip; IPG strip used was pH 4–7, 17 cm, linear; 12% polyacrylamide gel. a. Method A; b. Method B. (TIF) [file pone.0102175.s003.tif]

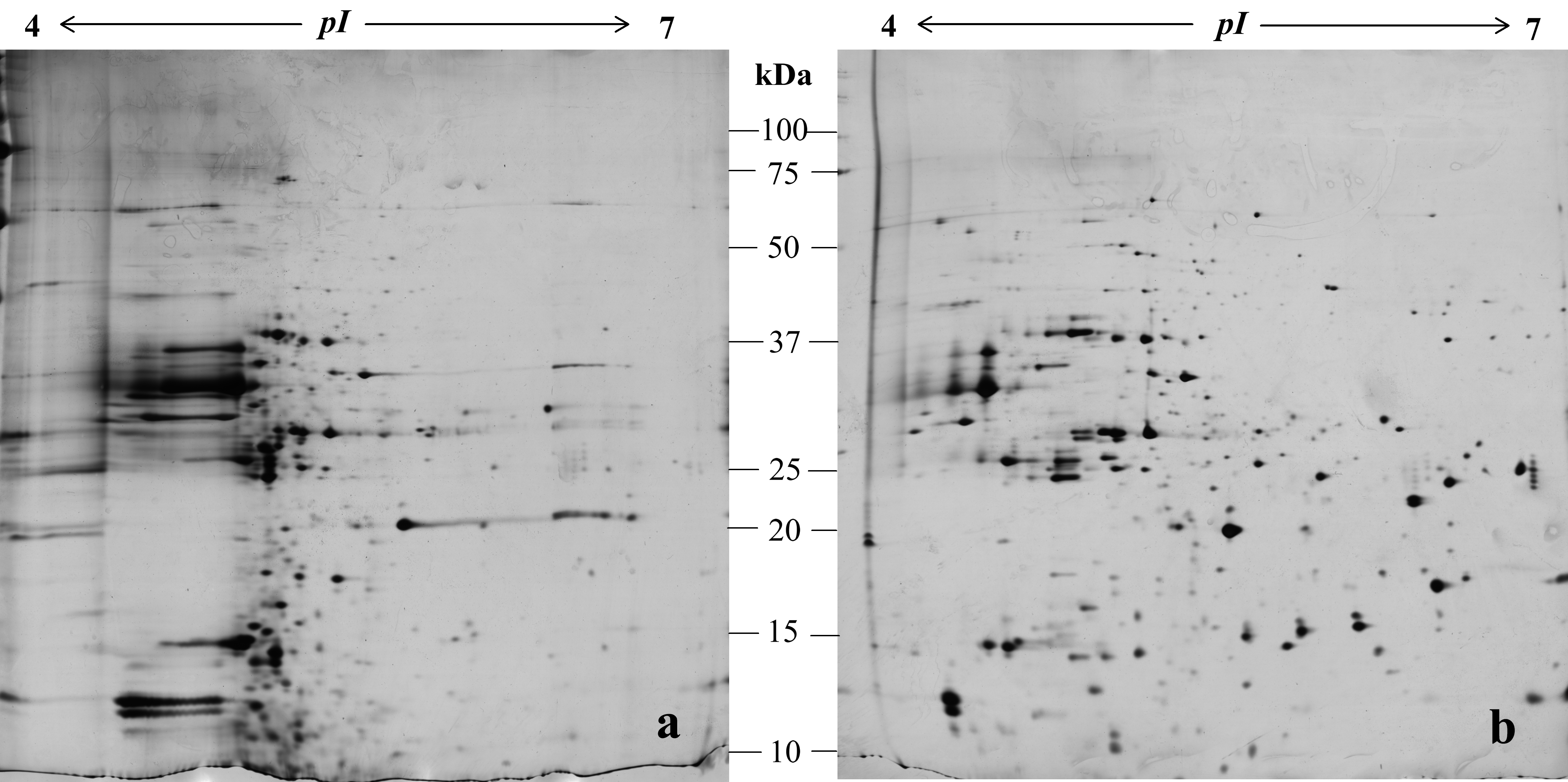

Supplement: Figure S4 — Two-dimensional electrophoretogram of proteins from mature leaves of pear. The sample loading amount was 150 µg/strip; IPG strip used was pH 4–7, 17 cm, linear; 12% polyacrylamide gel. a. Method A; b. Method B. (TIF) [file pone.0102175.s004.tif]

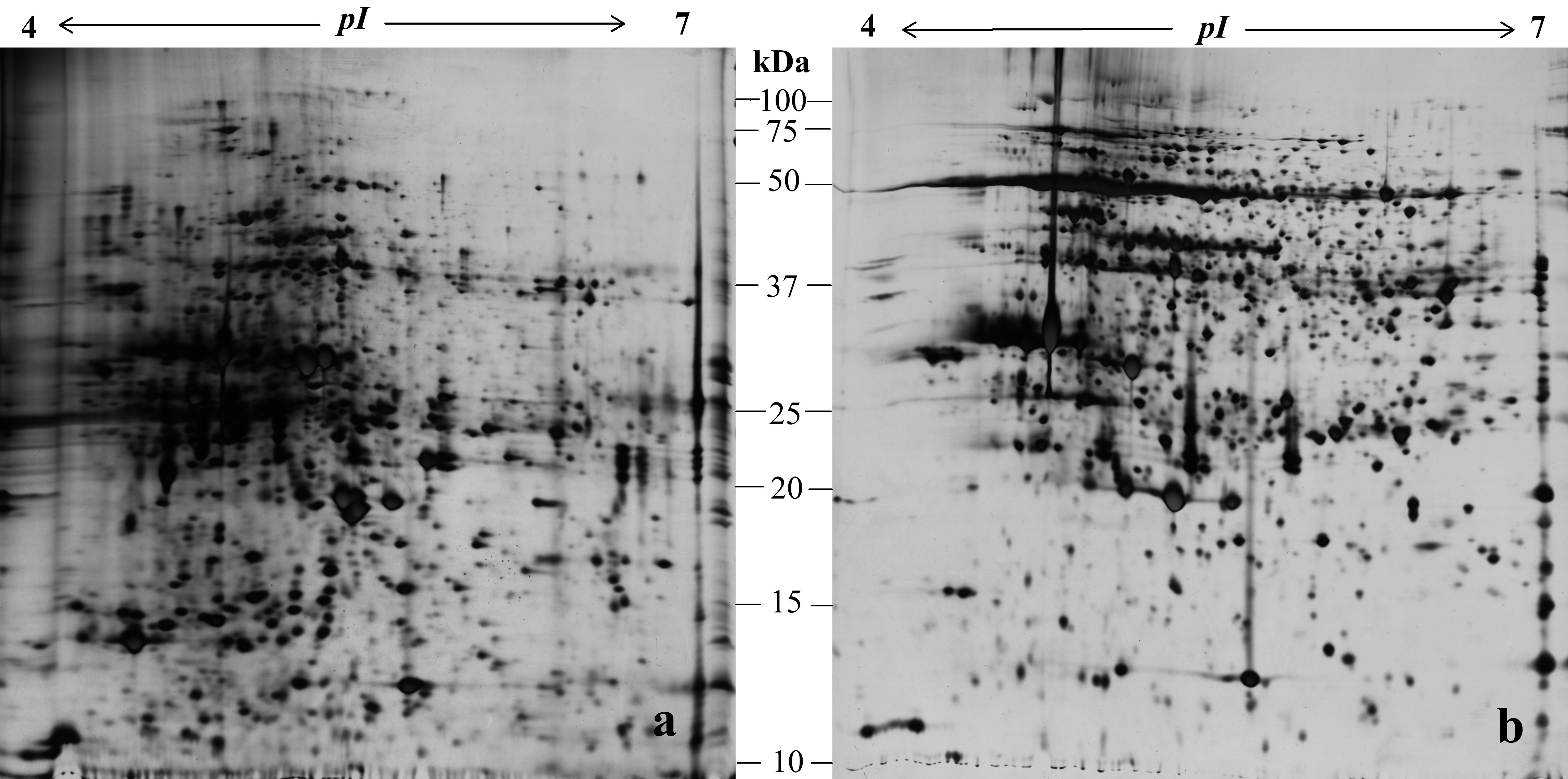

Supplement: Figure S5 — Two-dimensional electrophoretogram of proteins from mature leaves of orange. The sample loading amount was 150 µg/strip; IPG strip used was pH 4–7, 17 cm, linear; 12% polyacrylamide gel. a. Method A; b. Method B. (TIF) [file pone.0102175.s005.tif]

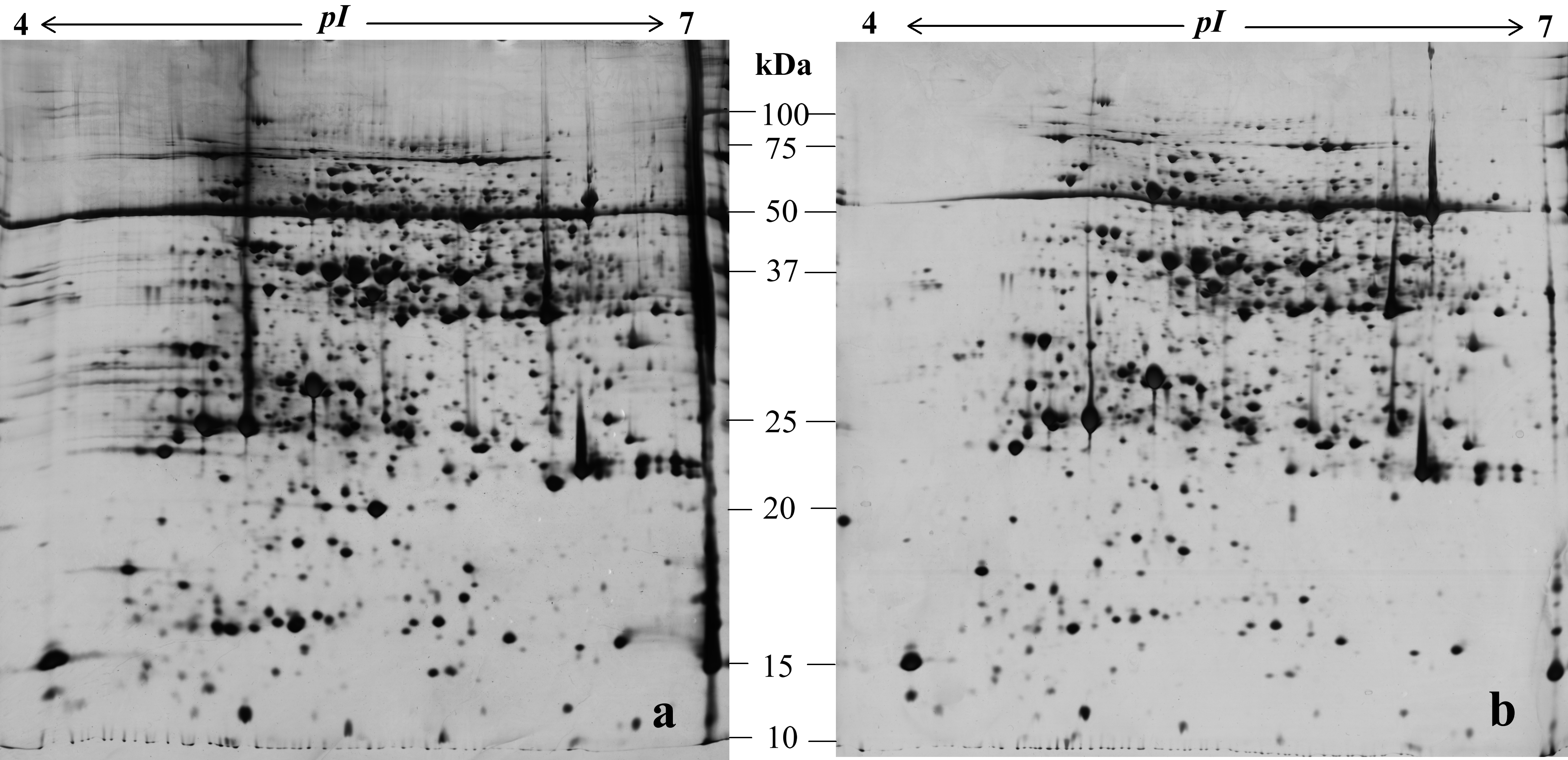

Supplement: Figure S6 — Two-dimensional electrophoretogram of proteins from mature leaves of ramie. sample loading amount was 150 µg/strip; IPG strip used was pH 4–7, 17 cm, linear; 12% polyacrylamide gel. a. Method A; b. Method B. (TIF) [file pone.0102175.s006.tif]
